# Synthesis and characterization of *meso*-substituted A_2_B corroles with extended π-electronic structure

**DOI:** 10.1007/s00706-017-2114-6

**Published:** 2017-11-29

**Authors:** Michael Haas, Sabrina Gonglach, Stefan Müllegger, Wolfgang Schöfberger

**Affiliations:** 1 Institute of Organic Chemistry, Johannes Kepler University Linz, Altenberger Straße 69, 4040 Linz, Austria; 2 Institute of Semiconductor and Solid State Physics, Altenberger Straße 69, 4040 Linz, Austria

**Keywords:** A_2_B corrole, Pyrene, Sila-Sonogashira coupling, π-Conjugation, Copper, Electron paramagnetic resonance

## Abstract

**Abstract:**

We report the chemical synthesis and characterization of the stable 5,15-bis(pentafluorophenyl)-10-(trimethylsilylethynyl)corrole which serves as a precursor for the subsequent in situ sila-Sonogashira-cross-coupling reaction and metalation with copper(II) acetate. Under ambient conditions and a common catalyst system the reaction with 1-iodopyrene occurred within five hours. Due to the direct conjugation of the 18π-electronic system of the corrole macrocycle over the alkynyl group to the pyrene moiety the optical transitions in the Soret (B-) band Q-band region are significantly altered. The copper corrole exhibited complex hyperfine and superhyperfine structure in the EPR spectrum. The assignment of the EPR spectrum reveals the existence of an axial [CuII-cor^∙+^] species.

**Graphical abstract:**

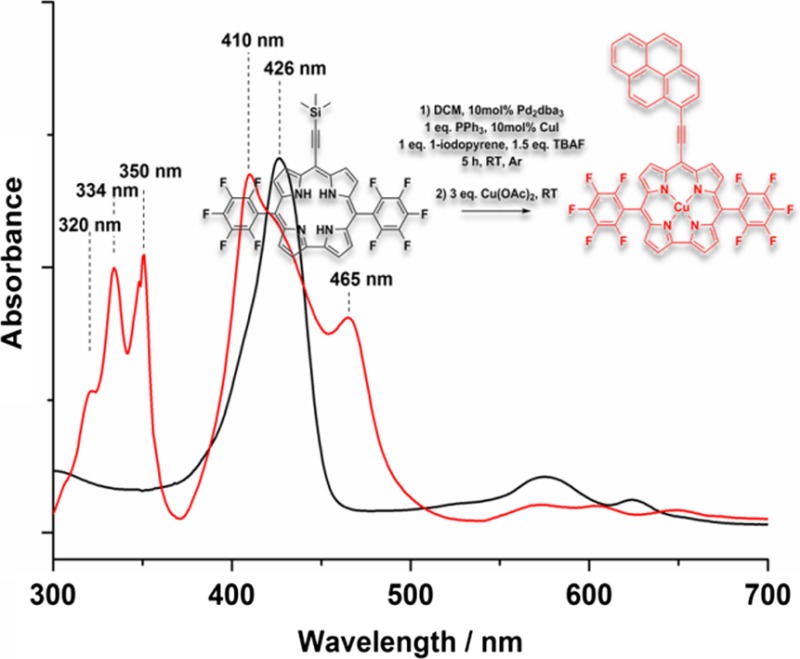

**Electronic supplementary material:**

The online version of this article (10.1007/s00706-017-2114-6) contains supplementary material, which is available to authorized users.

## Introduction

The establishment of novel synthesis routes towards symmetric and asymmetric corroles increased significantly over the past two decades [1–5]. This trend was triggered by the first published one-pot synthesis of corroles reported in 1999 by Gross and Paolesse [6, 7]. Since then, the scope of corrole application grew tremendously including examples in the fields of catalysis, photochemical sensors, molecular electronics, and biomedicinal applications [8–13]. For these purposes the availability of more sophisticated corrole systems is of great interest, which resulted in various contributions on corrole functionalization, including reactions on the β-pyrrolic positions such as bromination, hydroformylation, nitration, and chlorosulfonation as well as cycloadditions [3, 4]. Functionalizations on the *meso*-substituents include S_N_Ar-reactions and Buchwald–Hartwig type Pd-catalyzed aminations, and Pd-catalyzed C–C cross-coupling reactions (Suzuki–Miyaura and Liebeskind–Srogl) on metallated (Cu, Ag, Mn) corroles have also been reported [12, 14–18]. To sum up, a broad variety of synthesis protocols to obtain *meso*-substituted A_2_B and A_3_-corroles exist and are well applicable [4, 5, 16, 19]. The aromatic substituents at the *meso*-positions of the corrole macrocycle normally twist out of planarity with respect to the 18 π-electron macrocycle and consequently an extended π-electron conjugation to the *meso*-substituent is interrupted. Alkynyl groups as linker between the corrole core and the aromatic systems could prevent this behavior and would enlarge the π-electron conjugation. Recently, Osuka et al. presented a new methodology to synthesize *meso*-cumulenic 2*H*-corroles from a *meso*-ethynyl-3*H*-corrole as precursor [20].

In the underlying research work we discuss the chemical synthesis and functionalization of *meso*-trimethylsilyl-ethynyl-3H-corrole via in situ sila-Sonogashira cross-coupling reactions to extend the π-electron conjugation from the 18π-electron macrocycle over the alkynyl group to adjacent electron rich systems.

## Results and discussion

In this work, TMS-propynal was preferred as aldehyde for the corrole syntheses due to the high reactivity of propynal with nucleophiles and its tendency to form Michael addition products [21]. The first reaction step was the synthesis of 5-(pentafluoro)dipyrromethane (**1**) as precursor for the A_2_B-corrole synthesis. **1** was prepared according to the procedure of Dehean et al. [22] via condensation of pyrrole and pentafluorobenzaldehyde (Scheme 1) in water/HCl to give 89% yield. A prerequisite for further successful conversions is that pentafluorodipyrromethane **1** has to be purified via column chromatography and no other condensation product (tripyrromethane, bilane) must not be abundant.

**Figure:**
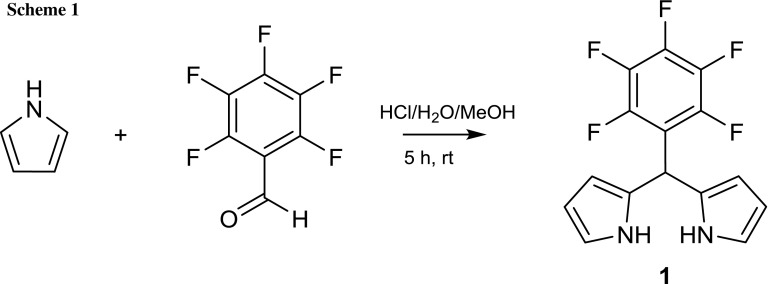

**Figure:**
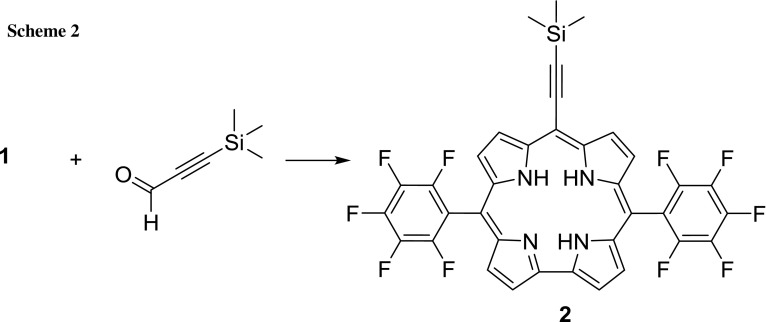

With the dipyrromethane precursor in hand we started the A_2_B corrole synthesis according to the water/MeOH method published by Gryko and Koszarna (Scheme 2)[23]. The important factor of a biphasic system is that small starting materials are well soluble in this medium, wherein long chain pyrrole products such as bilane precipitate and so undesired reactions with different pyrrole derivatives are suppressed [23, 24]. 2 Eq. of the dipyrromethane precursor **1** and 1 eq. of TMS-propynal were suspended in the biphasic water/MeOH system with 3 mol% HCl as catalyst. After complete consumption of the educts the mixture was extracted thrice with 20 cm^3^ DCM. The solution, which contains bilane and longer chain pyrrole products, was evaporated to dryness, dissolved with 40 cm^3^ DCM and subsequently treated with 1.25 eq. of DDQ. The oxidation reaction was performed at room temperature and quenched after 15 min by immediate evaporation of the solvent. The crude solid was purified via silica column chromatography (for further details—please see the “Experimental” section). Unfortunately the desired A_2_B-corrole was not obtained, instead H_3_TpFPC (5,10,15-tris(pentafluorophenyl)corrole) resulted preferred. This side reaction can be explained due to an rearrangement of adjacent pyrrole moieties via the published acidolysis mechanism by Lindsey et al., which is called “scrambling” [25, 26]. A proposed mechanism is shown in Scheme 3. In the aqueous acidic milieu the dipyrromethane **A** is protonated at one of the α-positions, of the pyrrolic system, and after electronic rearrangement, a pyrrole molecule is eliminated and **B** results. **B** combines with another dipyrromethane and forms tripyrromethane **C**, which can further react with another molecule of **B** to form bilane **D**. After oxidation with DDQ, H_3_TpFPC **3** is generated.

**Figure:**
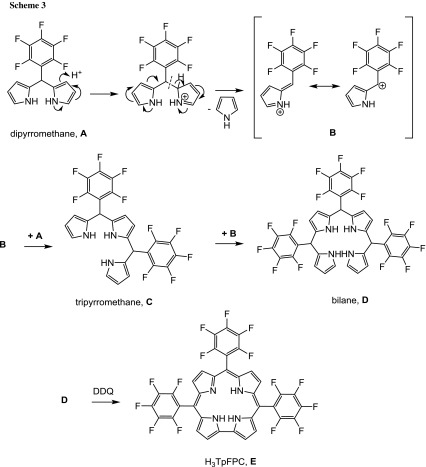

In contrast to our results, the group of Gryko synthesized A_2_B corroles without scrambling and high yields up to 54% [23]. The characteristic difference is, that they used moderate till high reactive aromatic aldehydes, instead of the non-aromatic TMS-propynal.

In 2015 Gryko et al. published a paper [24], where A_2_B *meso*-arylethynylporphyrins were synthesized via a biphasic system, which inspired us to try water/THF as solvent mixture. 5-(Pentafluorophenyl)dipyrromethane and TMS-propynal were suspended, HCl as catalyst added and the reaction mixture stirred for 2.5 h at room temperature. After extraction with DCM the solution was concentrated to 20 cm^3^ and DDQ was added. The oxidation was stopped after 15 min and purification via silica column chromatography followed. The best conditions obtained via screening the reaction was a solvent ratio of 2:1 (THF/water) and 0.37 eq. HCl as catalyst (Table 1). The water/co-solvent ratio is a key factor, since with higher water content organic intermediate derivatives are less soluble and precipitate preferred [24]. Due to this reason, we performed syntheses with a higher water/THF ratio (Table 1) with the expectation of preferred product **2** formation. Instead of higher yields, the opposite occurred and only traces of **2** could be obtained. There is no evident trend in the performed screening series, related to HCl concentration or water/THF ratio.

**Table 1:** Optimization of the biphasic synthesis of **2** (mM aldehyde/mM DPM)

Entry	HCl/eq.^a^	Water/THF ratio	H_3_TpFPC (scrambling)	Yield/%
1	0.0037	1:2	Yes	–
2	0.0185	1:2	Yes	–
3	0.037	1:2	Yes	–
4	0.37	1:2	No	5.7
5	0.37	1:1	Yes	Traces
6	0.37	2:1	Yes	Traces
7	0.37	5:1	Yes	Traces
8	0.37	10:1	Yes	Traces
9	3.7	1:2	Yes	–
10	37	1:2	No	5.5

All reactions were stirred for 10 min for the first step and later on diluted with DCM to 7.5 cm^3^ and oxidized with 2.8 eq. DDQ for 30 min

^a^Based on TMS-propynal

We tried to synthesize the A_2_B-corrole **2** using trifluoroactetic acid (TFA) as another Brönsted acid according to the synthetic procedure of Gryko [26]. Following, the same equivalents of dipyrromethane and aldehyde, DCM instead of the biphasic system discussed above and variation of the TFA concentration resulted in the same result and only H_3_TpFPC **3** was obtained (Table 2). This led us to diversify the temperature, using the same mol% TFA as described in the literature [27], where best yields were achieved at − 20 °C (Table 2). We accomplished the successful synthesis of desired pure A_2_B-corrole **2** within 15 min reaction time during the condensation step and further oxidation at room temperature with a reaction yield of 10.1%. Prolonging the reaction time yielded in the formation of **3** and other side-products. At − 78 °C no further improvement of the reaction yield was obtained.

**Table 2:** Screening of the reaction conditions using TFA as catalyst

Entry	*χ*(TFA)/mol%^a^	*T*/°C	Reaction time	H_3_TpFPC (scrambling)	Yield/%
1	8.5	RT	4 h	Yes	–
2	8.5	0	4 h	Yes	–
3	8.5	− 10	4 h	Yes	–
4	8.5	− 20	4 h	No	2.2
5	12.2	− 20	4 h	Yes	–
6	10.5	− 20	1 h	Yes	–
7	6.4	− 20	1 h	Yes	–
8	5.7	− 20	1 h	Yes	–
9	1.3	− 20	1 h	Yes	–
10	1.3	− 20	5 min	No	2.0
11	1.3	− 20	15 min	No	3.2
12	0.5	− 20	5 min	No	6.3
13	0.5	− 20	15 min	No	10.1
14	0.5	− 70	15 min	No	2.5

All reactions were performed under the following conditions: (1) Step: 2 eq. (*c*(DPM) = 133 mM), 1 eq. (*c*(TMS-Propynal) = 66 mM); (2) Step: 2.5 eq. DDQ

^a^Based on DPM

Inspired by the published synthetic procedure of Shaikh et al. [28], for a solvent free synthesis of dipyrromethanes with SnCl_2_·2H_2_O as catalyst, in the next step, we decided to use this method to synthesize corrole **2** with a series of Lewis acid catalysts.

First we varied the loading of the catalyst from equimolar to 0.04 equivalents with respect to the TMS-propynal. 0.64 mmol of DPM, 0.32 mmol TMS-propynal, and SnCl_2_·2H_2_O (0.04–1.0 eq.) were stirred in a 100 cm^3^ round-flask at room temperature for 10 min (Table 3). A black powder was formed, which was further dissolved in 7.5 cm^3^ DCM, 2.8 eq. DDQ were added portion wise and the solution was allowed to stir for 30 min. After common work-up the highest yield could be achieved using 0.04 eq. (Table 3) of the catalyst. With rising catalyst loadings the formation of H_3_TpFPC **3** is preferred, in contrast to the desired product **2**. Entries 3–6 (Table 3) show four more Lewis acids which were investigated, due to their catalytic properties for the proper activation of the aldehyde. However, the syntheses were without success and again only H_3_TpFPC **3** was formed (Table 4).

**Table 3:** Screening of the reaction conditions using SnCl_2_·2H_2_O as catalyst

Entry	*n*(DPM)/mmol	*n*(Ald.)/mmol	SnCl_2_·2H_2_O eq.^a^	H_3_TpFPC	Yield/%
1	0.64	0.32	0.04	No	1.7
2	0.64	0.32	0.28	Yes	1.1
3	0.64	0.32	0.40	Yes	–
4	0.64	0.32	1.00	Yes	–

All reactions were stirred for 10 min for the condensation step and later on diluted with DCM to 7.5 cm^3^ and oxidized with 2.8 eq. DDQ for 30 min

^a^Based on TMS-Propynal

**Table 4:** Varying the used acid for the solvent free synthetic procedure

Entry	*n*(DPM)/mmol	*n*(Ald.)/mmol	Acid^a^	H_3_TpFPC	Yield/%
1	0.32	0.16	BF_3_ · Et_2_O	Yes	–
2	0.32	0.16	TFA	Yes	–
3	0.32	0.16	InCl_3_	Yes	–
4	0.32	0.16	Gd(OTf)_3_	Yes	–
5	0.32	0.16	Cu(OTf)_2_	Yes	–
6	0.32	0.16	Sc(OTf)_3_	Yes	–

All reactions were stirred for 10 min for the first step and later on diluted with DCM to 7.5 cm^3^ and oxidized with 2.8 eq. DDQ for 30 min

^a^0.04 eq. based on TMS-Propynal

In contrast to the results of Gryko et al. [19] and Osuka et al. [20] the deprotection of **2** occurred within few minutes with 1.5 eq. of TBAF (Scheme 4) and was determined via mass spectroscopy and UV–Vis measurements (see electronic supporting information). The isolation of the desilylated corrole **3** was more problematic due to its behaviour to decompose, which could be observed in ^1^H NMR in which signals in the range of 6–7 ppm were detected stemming from open-chain structure(s).

**Figure:**
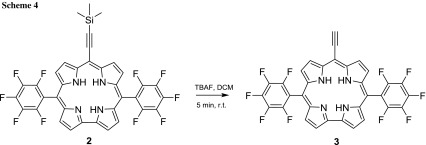

**Fig. 1:**
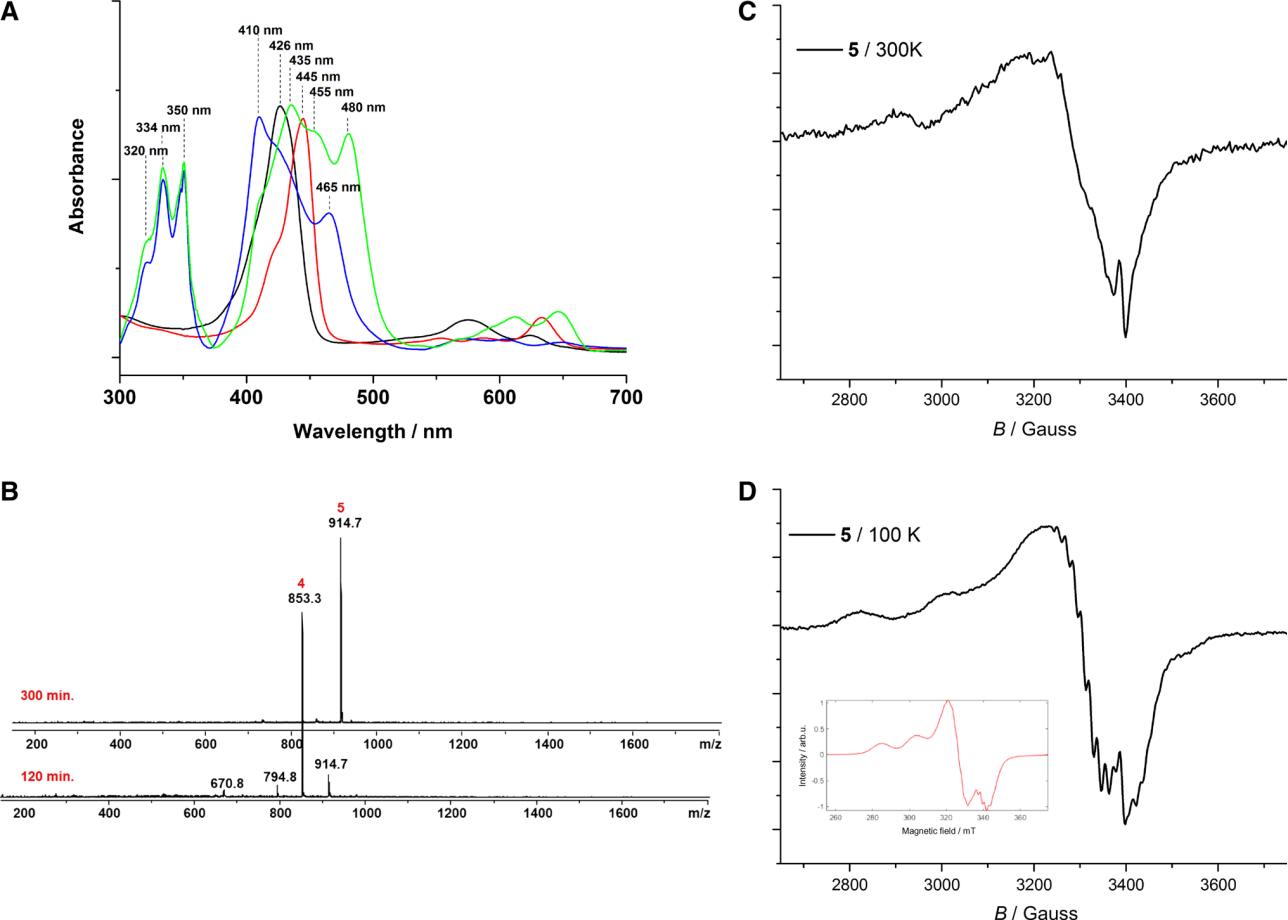
**a** UV–Vis spectral and **b** mass spectrometric analysis during the one-pot sila-Sonogoshira of **2** (black line) to compound **4** (green line) and subsequent metalation with Cu(OAc)_2_ at room-temperature to the copper corrole **5** (blue line). **c**, **d** EPR spectra of **5** in chloroform at 300 K and 100 K. Simulated spectrum (inset, **d**)

Consequently, further functionalisation of the corrole had to be done via a one-pot synthesis procedure starting from corrole **2**. In this endeavour we decided to expand the π-system using sila-Sonogashira cross-coupling conditions to ensure decomposition of the corrole precursor would not occur (Scheme 5). Hence, 1 eq. of **2** was mixed with 1.5 eq. TBAF, 1 eq. 1-iodopyrene, 0.1 eq. Pd_2_dba_3_, 0.1 eq. CuI, and 1 eq. PPh_3_ in TEA/DCM (1:2) and was allowed to stir until no deprotected corrole **3** was observed in mass spectrum (Fig. SI and Fig. 1). Within 5 h reaction time the same reaction solution was treat with Cu(OAc)_2_ and after conventional work-up the corrole **5** was obtained with 57% yield.

**Figure:**
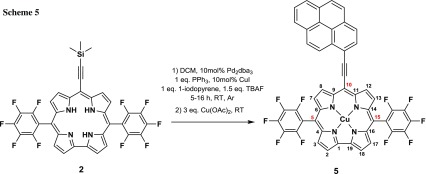

Figure 1a, b exhibits the UV–Vis spectral and mass spectral changes during the reaction of corrole **2** via the desilylation process leading to corrole **3** and the subsequent conversion to the free-base 5,15-bis(pentafluorophenyl)-10-(pyreneethynyl)corrole **4** and finally the metalation with Cu(OAc)_2_ to the product **5**. Strong shifts and splitting of the Soret band maxima of free-base 5,15-bis(pentafluorophenyl)-10-(pyreneethynyl)corrole **4** and the according copper corrole **5** are visible and suggest the strong coupling of the π-electron system of pyrene over the triple-bond to the 18 π-electronic system of the corrole. Moreover, the rather broad lines in the ^1^H and ^19^F NMR spectra (see Fig. S9 and Fig. S11) indicate at least partial contribution of *d* ^9^-species with Cu(II)corrole radical cation character, which can adopt either an antiferromagnetically coupled singlet state (saddle-shape geometry) or a planar triplet state [29]. Variable temperature EPR spectra of **5** were recorded in chloroform at 300 and 100 K and the corresponding spectra are shown in Fig. 1c, d, respectively. A square planar Cu^III^ complex would be diamagnetic in nature while a Cu^II^ corrole should show an axial doublet for a single unpaired electron (S = 1/2) with hyperfine coupling of ^65^Cu/^63^Cu (I = 3/2) to give a four line spectrum. However, this signal is then further split by ^14^N (I = 1) to give a nine line spectral pattern. Indeed, defined EPR signals are observed for **5** with metal hyperfine (< 3100 Gauss) and ligand super-hyperfine interactions at low temperature, which accounts for the existence of [CuII-cor^∙+^] with the expected *g*∥ (or *g*_z_) = 2.173, *g*⊥ (or *g*_*x,y*_) = 2.07, and *A*∥ = 19.3 mT (= 541 MHz) as shown in Fig. 1c, d (simulated spectrum illustrated as an insert in Fig. 1d) and, therefore, the non-innocence character of the corrole macrocycle. Electrochemical and spectrochemical investigations are in progress and beyond the scope of the underlying work.

## Conclusion

We have established a novel synthesis procedure to obtain the 5,15-bis(pentafluorophenyl)-10-(trimethylsilylethynyl)corrole. This *meso*-substituted A_2_B corrole could not be synthesized via a standard procedure due to the competing fast scrambling process during the reaction. Successful conversion of the less reactive TMS-propynal (compared to the commonly employed arylaldehydes) with the dipyrromethane to the corrole was only obtained by the following the reaction conditions: 2 eq. DPM, 1 eq. TMS-propynal, and 0.5 mol% TFA (based on DPM) at − 20 °C (synthetic procedure A). The desired product was obtained in 10% yield. The stable 5,15-bis(pentafluorophenyl)-10-(trimethylsilylethynyl)corrole serves as a precursor for the subsequent functionalization via sila-Sonogashira-cross-coupling reaction with 1-iodopyrene under ambient conditions and a common catalyst system and further metalation with Cu(OAc)_2_. This functionalization procedure leads to an A_2_B corrole with an extended π-electronic structure at *meso*-position 10. The obtained free-base and copper corroles reveal strong changes in the UV–Vis spectral region.

## Experimental

All chemicals were purchased from Alfa Aesar, Fluka, Merck, or Sigma-Aldrich and used without further purification. THF was distilled over sodium and benzophenone under an argon atmosphere and stored over molecular sieve (4 Å) until use. DCM was distilled over P_2_O_5_ under an argon atmosphere and stored over molecular sieve (4 Å) upon use. All solvents for the NMR were purchased from Euriso-Top. TLC was performed on Macherey–Nagel silica gel 60 (0.20 mm) with fluorescent indicator UV_254_ on aluminium plates and on Merck aluminium oxide 60 (0.20 mm) with fluorescent indicator UV_254_ on aluminium plates. For chromatography, silicagel columns were prepared with silicagel 60 (0.070–0.20 mesh) from Grace and aluminium oxide columns were prepared with aluminium oxide (activated, basic, Brockmann I) from Sigma-Aldrich. Proton (^1^H NMR) and Carbon (^13^C NMR) spectrum were recorded on a Bruker Ascend 700 MHz Avance III NMR spectrometer and on a Bruker Avance 300 MHz NMR spectrometer. Fluor (^19^F NMR) spectra were recorded on a Bruker Avance 300 MHz NMR spectrometer at 282.4 MHz. The chemical shifts are given in parts per million (ppm) on the delta scale (*δ*) and are referred to the used deuterated solvent for ^1^H NMR and to TFA for ^19^F NMR. Mass spectra were measured on a Finnigan LCQ DecaXPplus Ion trap mass spectrometer with ESI ion source and HRMS was performed on a 6510 quadrupole/time-of-flight (Q-TOF) instrument (Agilent). UV–Vis absorption spectra were collected on a Varian CARY 300 Bio spectrophotometer from 200 to 900 nm. Fluorescence spectra were measured on a CARY eclipse fluorescence spectrophotometer.

### 5-(Pentafluorophenyl)dipyrromethane (**1**)

Synthesis according to the procedure of Dehean et al. [22], 2.12 cm^3^ of freshly distilled pyrrole (30.6 mmol, 3 eq.) and 2.00 g of pentafluorobenzaldehyde (10.2 mmol, 1 eq.) were mixed with 100 cm^3^ 0.18 M HCl and stirred for 5 h at room temperature under N_2_. After 5 h the precipitated product was filtered off and washed with water and heptane. Yield: 2.46 g (7.8 mmol, 77%); ^1^H NMR (300 MHz, CDCl_3_, 25 °C): *δ* = 8.20 (bs, 2H, NH), 6.75 (m, 2H, pyrrole-H), 6.19 (m, 2H, pyrrole-H), 6.05 (m, 2H, pyrrole-H), 5.92 (bs, 1H, meso-H) ppm; ^19^F NMR (300 MHz, CDCl_3_, 25 °C): *δ* = − 141.48 to − 141.42 (d, *J*(F,F) = 18 Hz, 2F, *ortho*-F), − 155.66 to − 155.81 (t, *J*(F,F) = 21 Hz, 1F, *para*-F), − 161.20 (m, 2F, *meta*-F) ppm; MS(ESI^+^): *m/z* calcd. for C_15_H_9_F_5_N_2_ 312.07, found 313.0 ([M+H]^+^).

### 5,15-Bis(pentafluorophenyl)-10-(trimethylsilylethynyl)corrole (**2**, C_36_H_20_F_10_N_4_Si)

*Method A*: 47.3 mm^3^ of TMS-propynal (0.32 mmol, 1 eq.) and 100 mm^3^ of the TFA-solution (2.4 mm^3^ TFA in 1 cm^3^ CH_2_Cl_2_) were stirred under N_2_ atmosphere for 5 min in 2.0 cm^3^ CH_2_Cl_2_ at − 20 °C. Dropwise addition of 200 mg of **1** (0.64 mmol, 2 eq.) dissolved in 2.7 cm^3^ CH_2_Cl_2_ over 5 min followed. The reaction mixture was then stirred for 15 min at − 20 °C under N_2_ atmosphere. After dilution with CH_2_Cl_2_ to 7.2 cm^3^ and portion wise addition of 94.51 mg DDQ (0.42 mmol, 1.3 eq.), the solution was stirred for 20 min at room temperature under N_2_ atmosphere. Evaporation of the solvent and purification via column chromatography (silica, CH_2_Cl_2_/heptane 1:1) followed up. All fluorescent bands were collected and the solvent was evaporated to dryness. Yield: 23.5 mg (0.032 mmol, 10.1%).

*Method B*: A suspension containing 29.6 mm^3^ of TMS-propynal (0.20 mmol, 1 eq.), 2.5 cm^3^ THF, 1.25 cm^3^ H_2_O, and 0.6 mm^3^ HCl_conc_ was stirred under Ar atmosphere for 5 min at room temperature. Portion wise addition of 125 mg of **1** (0.40 mmol, 2 eq.) followed. The solution was stirred for 3 h under Ar atmosphere at room temperature. The mixture was extracted with CH_2_Cl_2_, the organic phase dried over Na_2_SO_4_ and concentrated. The collected brownish solid was dissolved in 45 cm^3^ CH_2_Cl_2_ and 59 mg DDQ (0.26 mmol, 1.3 eq.) added. After 30 min stirring at room temperature the solution was concentrated and purification via column chromatography (silica, CH_2_Cl_2_/heptane 1:1) followed. All fluorescent bands were collected and the solvent was evaporated to dryness. Yield: 8.2 mg (0.011 mmol, 5.7%).

*Method C*: 23.67 mm^3^ TMS-propynal (0.16 mmol, 1 eq.), 100 mg of **1** (0.32 mmol, 2 eq.), and 0.04 eq. of the Lewis acid were combined and stirred for 15 min at room temperature. Throughout the stirring the viscous mixture turned into a black powder, which was further dissolved in 7.5 cm^3^ DCM. DDQ (2.8 eq.) was added portion wise and the solution was allowed to stir at room temperature. After 30 min stirring at room temperature the solution was concentrated and purification via column chromatography (silica, CH_2_Cl_2_/heptane 1:1) followed. All fluorescent bands were collected and the solvent was evaporated to dryness. Yield: 2.0 mg (0.003 mmol, 1.7%).

^1^H NMR (300 MHz, CDCl_3_, 25 °C): *δ* = 9.38–9.40 (d, *J*(H,H) = 4.8 Hz, 2H, pyrrole-H), 9.00–9.02 (d, *J*(H,H) = 4.3 Hz, 2H, pyrrole-H), 8.71–8.73 (d, *J*(H,H) = 4.8 Hz, 2H, pyrrole-H), 8.47–8.48 (d, *J*(H,H) = 4.1 Hz, 2H, pyrrole-H), 0.59 (bs, 9H, CH_3_) ppm; ^19^F NMR (300 MHz, CDCl_3_, 25 °C): *δ* = −141.48 to − 141.42 (d, *J*(F,F) = 18 Hz, 2F, *ortho*-F), − 155.66 to − 155.81 (t, *J*(F,F) = 21 Hz, 1F, *para*-F), -161.20 (m, 2F, *meta*-F) ppm; MS(ESI^−^): *m/z* calcd. for C_36_H_20_F_10_N_4_Si ([M-H]^+^) 725.1209, found 727.1219; UV/Vis (CH_2_Cl_2_): *λ*_max_ (*ε*) = 427 (7.93 × 10^3^), 575 (1.85 × 10^3^), 624 (1.37 × 10^3^) nm (dm^3^ mol^−1^ cm^−1^).

### Copper 5,15-bis(pentafluorophenyl)-10-(pyreneethynyl)corrole (**5**, C_49_H_17_CuF_10_N_4_)

10 mg TMS-ethynylcorrole **1** (0.013 mmol) was dissolved in 3.2 cm^3^ DCM and 1.9 cm^3^ TEA. The solution was purged with argon and several freeze–pump–thaw cycles followed. 5.3 mg TBAF (0.020 mmol, 1.5 eq.), 4.5 mg Pd_2_dba_3_ (0.013 mmol, 1 eq.), 10 mol% CuI, and 10 mol% PPh_3_ were added and the resulting mixture was stirred at room temperature under argon overnight. After completion of the reaction, 7 mg Cu(OAc)_2_ (0.039 mmol, 3.3 eq.) were added the reaction was allowed to stir for another hour. The crude was filtered through Celite and then washed by extraction with water and dichloromethane. The organic phase was dried over Na_2_SO_4_ and concentrated. Purification was accomplished via column chromatography (silica, CH_2_Cl_2_/heptane 5:1). Yield: 7.1 mg (0.008 mmol, 57%). ^1^H NMR (300 MHz, CDCl_3_, 25 °C): *δ* = 8.49 (bs, 1H, pyrrole-H), 8.28 (bs, 1H, pyrrole-H), 8.19 (m, 2H, pyrrole-H), 8.13 (m, 1H, pyrrole-H), 8.01 (bs, 2H, pyrrole-H), 7.85 (bs, 1H, pyrrole-H), 7.71 (bs, 1H, pyrene-H), 7.61 (bs, 4H, pyrene-H), 7.53 (bs, 1H, pyrene-H), 7.44 (bs, 1H, pyrene-H), 7.40 (bs, 2H, pyrene-H) ppm; ^19^F NMR (300 MHz, CDCl_3_, 25 °C): *δ* = -137.67 (m, 4F, *ortho*-F), -152.22 (m, 2F, *para*-F), -161.43 (m, 4F, *meta*-F) ppm; MS(ESI^−^): *m/z* calcd. for C_49_H_17_F_10_N_4_Cu 914.0590, found 914.0549 ([M−H]^+^); UV/Vis (CH_2_Cl_2_): *λ*_max_ (*ε*) = 269 (5.66 × 10^3^), 280 (6.76 × 10^3^), 319 (5.53 × 10^3^), 334 (7.02 × 10^3^), 351 (7.09 × 10^3^), 409 (7.39 × 10^3^), 426 (6.85 × 10^3^), 464 (5.41 × 10^3^), 570 (2.74 × 10^3^), 609 (2.33 × 10^3^), 645 (1.88 × 10^3^) nm (dm^3^ mol^−1^ cm^−1^).

## Electronic supplementary material

Below is the link to the electronic supplementary material.

Supplementary material 1 (DOCX 1468 kb)
