# Supplementary material for: Synthesis and characterization of meso-substituted A2B corroles with extended π-electronic structure
Source: Monatsh Chem. 2017 Nov 29;149(4):773–81. doi: 10.1007/s00706-017-2114-6 (PMC5906495; doi:10.1007/s00706-017-2114-6)
Supplement: Supplementary file 1 — Supplementary material 1 (DOCX 1468 kb) [file 706_2017_2114_MOESM1_ESM.docx]

**Supporting Information for**

Synthesis and characterization of *meso-*substituted A_2_B corroles with extended π−electronic structure

Sabrina Gonglach^1^, Michael Haas^1^, Stefan Müllegger^2^, Wolfgang Schöfberger^2,🖂^

*Institute of Organic Chemistry, Johannes Kepler University Linz (JKU), Altenberger Straße 69, 4040 Linz, Austria.*

*Institute of Semiconductor and Solid State Physics, Altenberger Straße 69, 4040 Linz, Austria.*

🖂 Wolfgang Schöfberger

*E-mail:* [*wolfgang.schoefberger@jku.at*](mailto:wolfgang.schoefberger@jku.at)

## General Information

All chemicals were purchased from Alfa Aesar, Fluka, Merck or Sigma-Aldrich and used without further purification. Dipyrromethane was synthesized according to literature ^[1]^. THF was distilled over sodium and benzophenone under an argon atmosphere and stored over molecular sieve (4 Å) until use. DCM was distilled over P_2_O_5_ under an argon atmosphere and stored over molecular sieve (4 Å) upon use. All solvents for the NMR were purchased from Euriso-Top. TLC was performed on Macherey-Nagel silica gel 60 (0.20 mm) with fluorescent indicator UV_254_ on aluminium plates and on Merck aluminium oxide 60 (0.20 mm) with fluorescent indicator UV_254_ on aluminium plates. For chromatography, silica-gel columns were prepared with silica-gel 60 (0.070 – 0.20 mesh) from Grace and aluminium oxide columns were prepared with aluminium oxide (activated, basic, Brockmann I) from Sigma-Aldrich. Proton (^1^H-NMR) and Carbon (^13^C-NMR) spectra were recorded on a Bruker Ascend 700 MHz Advance III NMR spectrometer and on a Bruker Advance 300 MHz NMR spectrometer. Fluor (^19^F-NMR) spectra were recorded on a Bruker Advance 300 MHz NMR spectrometer at 282.4 MHz. The chemical shifts are given in parts per million (ppm) on the delta scale (δ) and are referred to the used deuterated solvent for ^1^H-NMR and to TFA for ^19^F-NMR. Mass spectra were measured on a Finnigan LCQ DecaXPplus Ion trap mass spectrometer with ESI ion source and HRMS was performed on a 6510 quadrupole/time-of-flight (Q-TOF) instrument (Agilent). UV-Vis absorbtion spectra were collected on a Varian CARY 300 Bio spectrophotometer from 200 to 900 nm. Fluorescence spectra were measured on a CARY eclipse fluorescence spectrophotometer.

## Characterization of 5, 15-pentafluorophenyl-10-trimethylsilylethynylcorrole


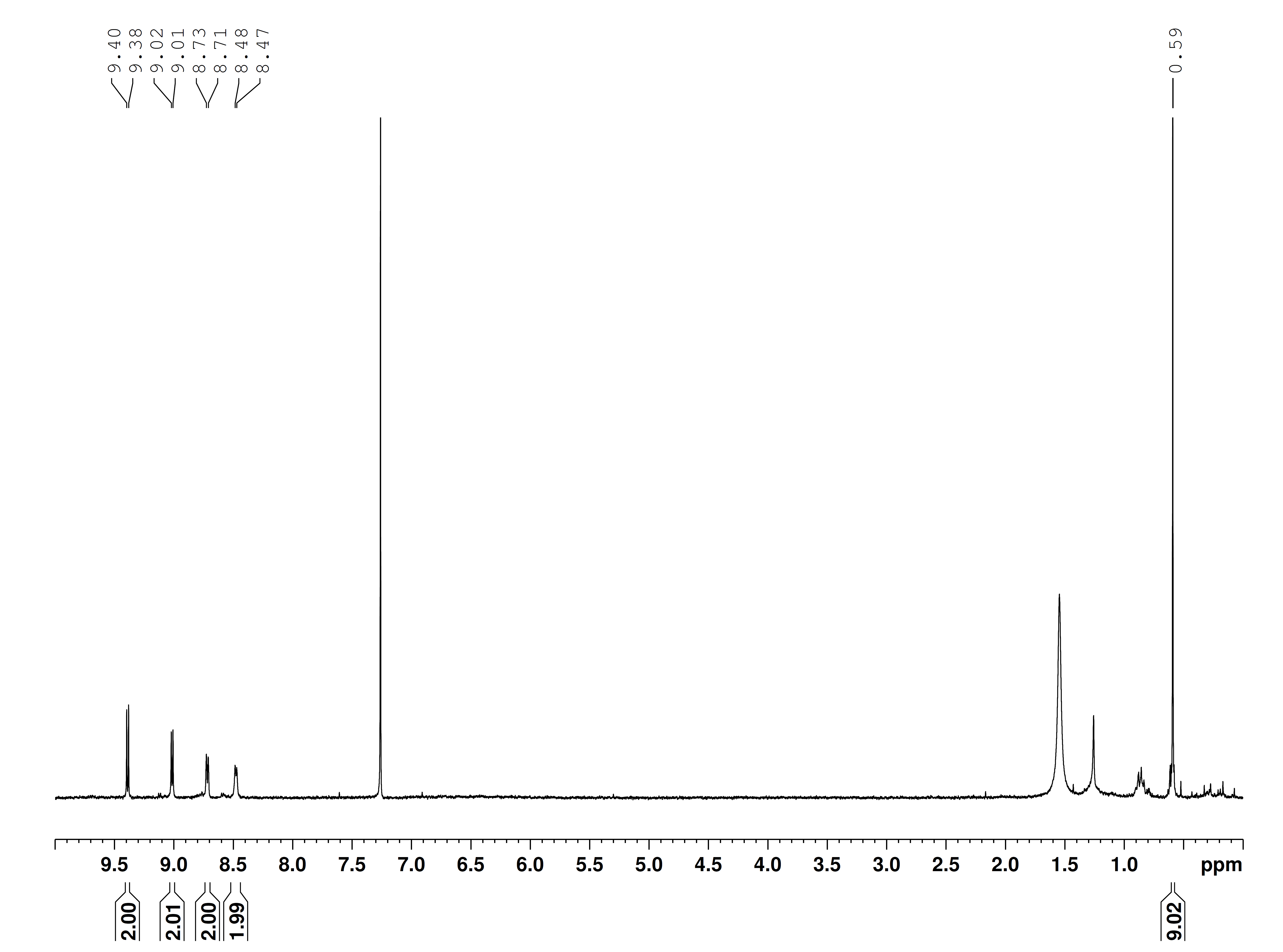


Fig.S1: ^1^H-NMR spectrum of 5, 15-pentafluorophenyl-10-trimethylsilylethynyl-corrole 2.


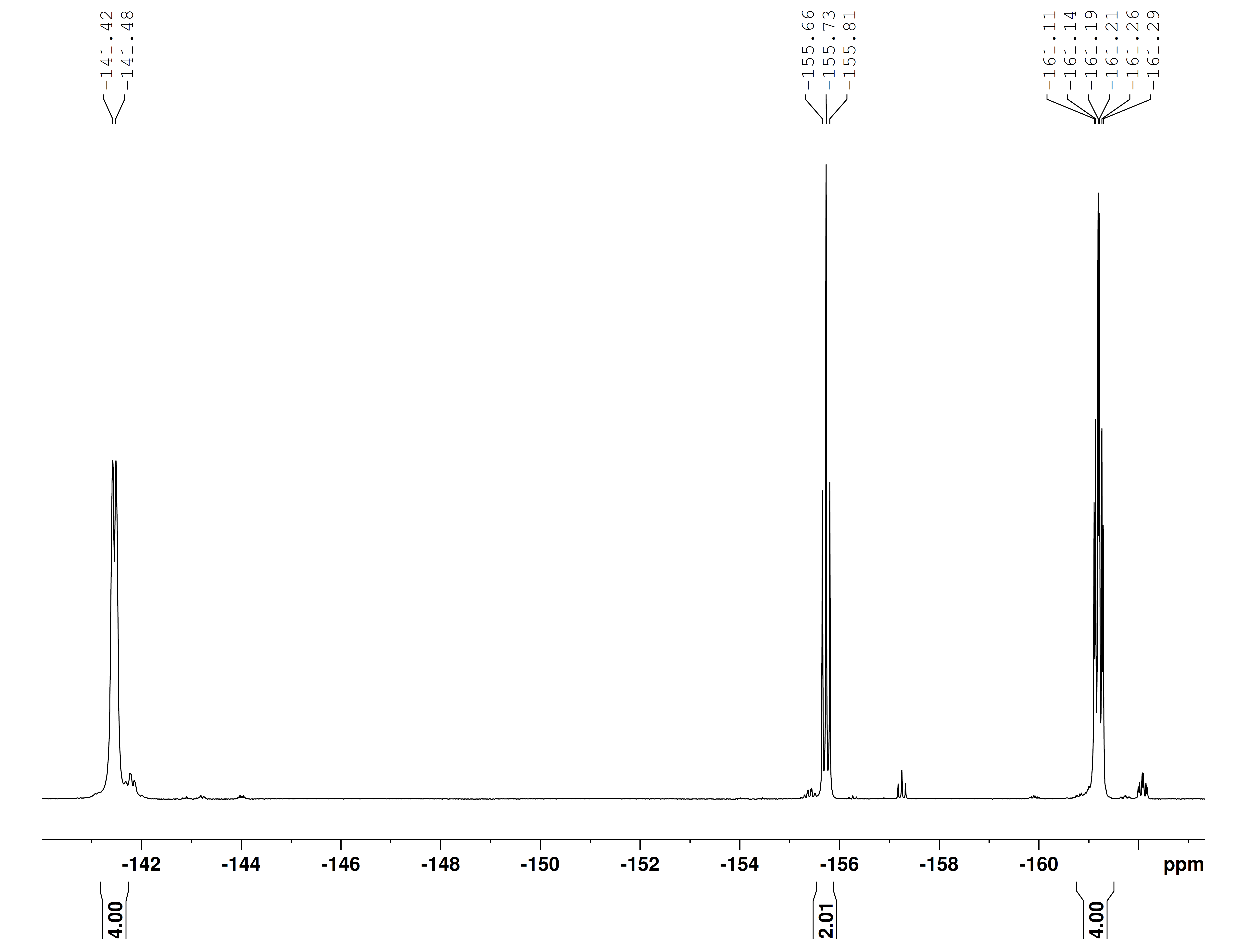


Fig. S2: ^19^F-NMR spectrum of 5, 15-pentafluorophenyl-10-trimethylsilylethynyl-corrole 2.


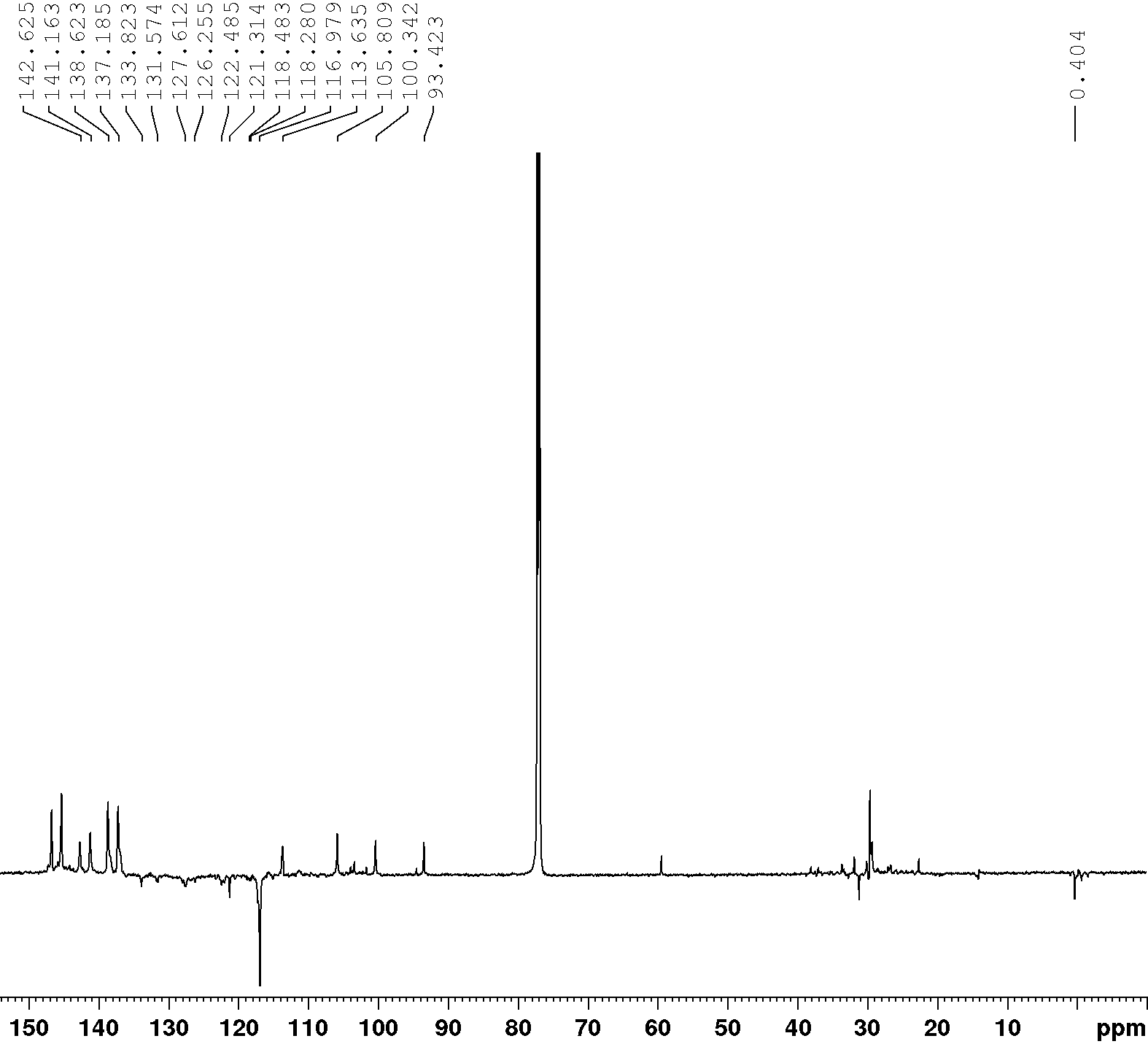


Fig. S3: ^13^C-NMR spectrum of 5, 15-pentafluorophenyl-10-trimethylsilylethynyl-corrole **2** (attached proton test - APT).

***
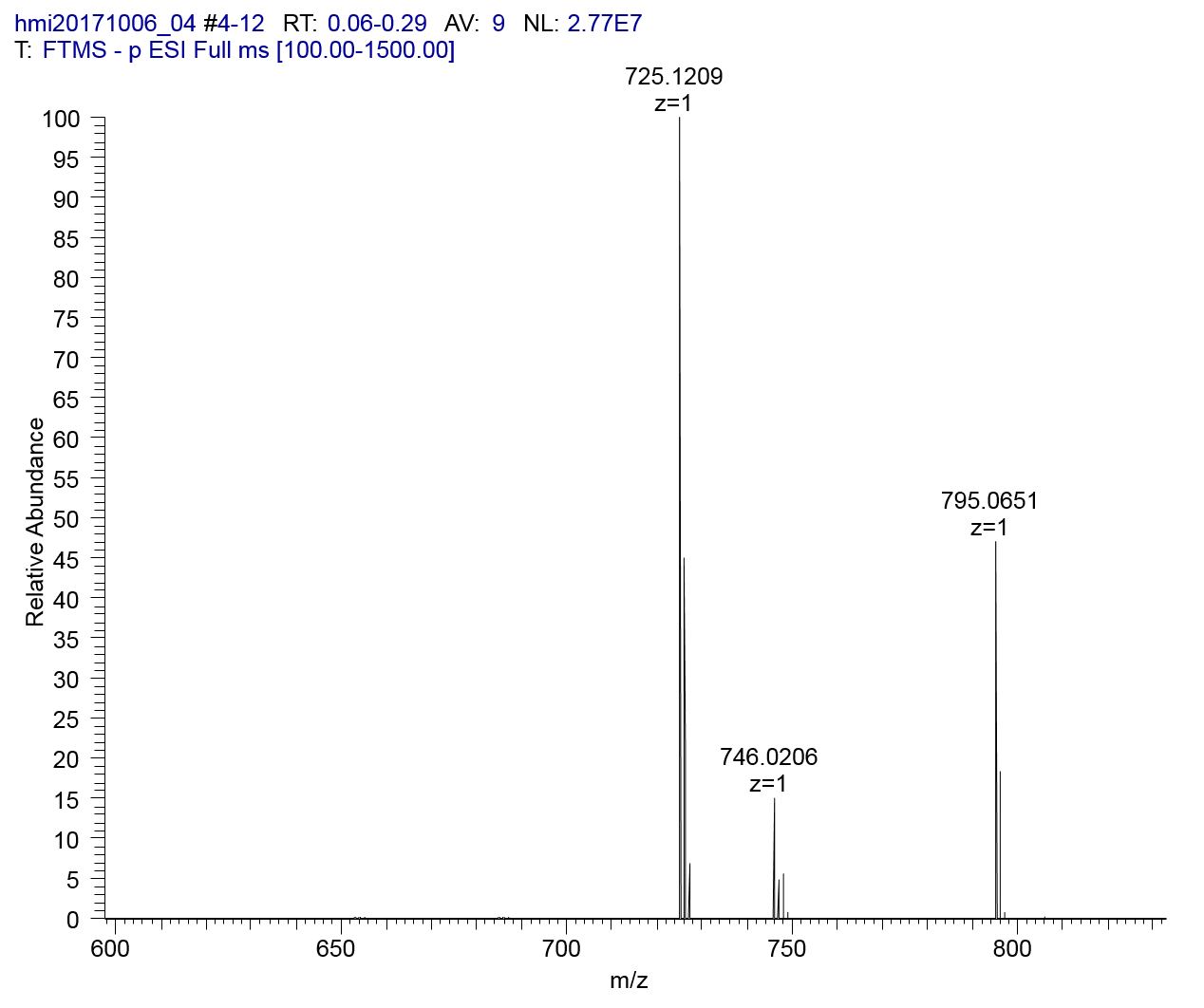
***

H_3_TpFPC

Fig. S4: HRMS of 5, 15-pentafluorophenyl-10-trimethylsilylethynyl-corrole **2**.

**
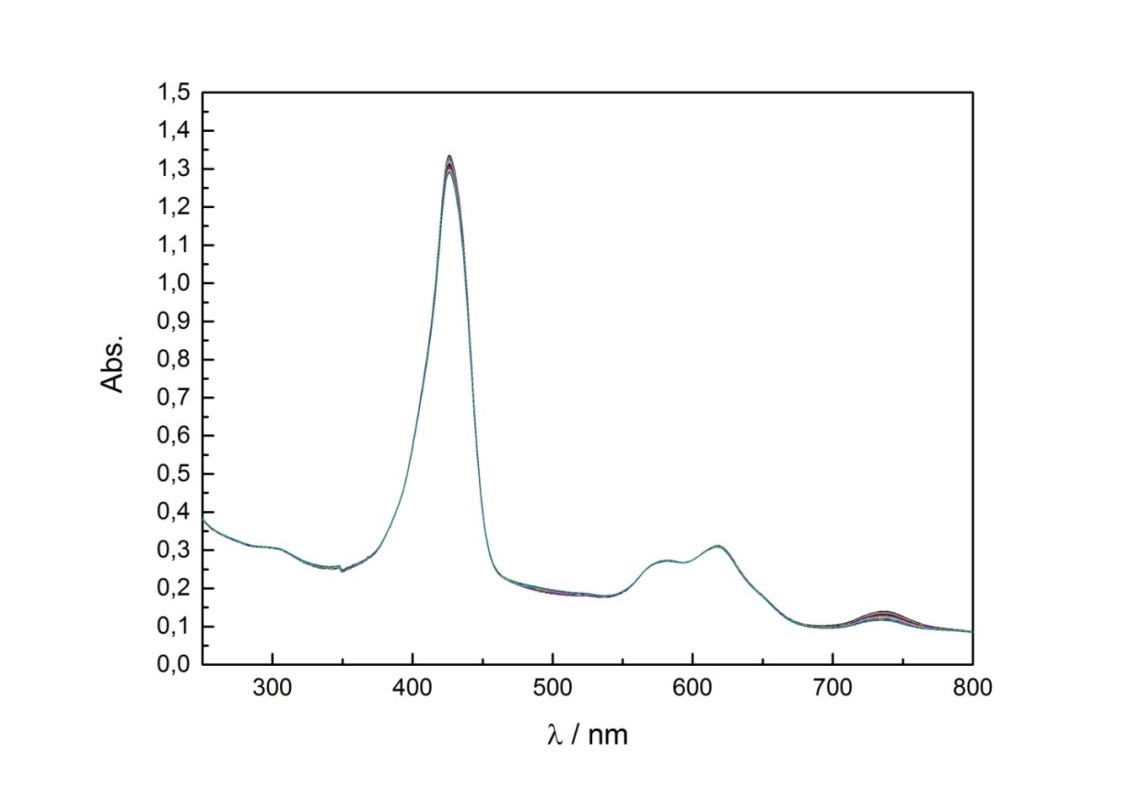
**

Fig. S5: UV-vis spectra of the stability test of 5, 15-pentafluorophenyl-10-trimethyl silylethynyl-corrole **2**.


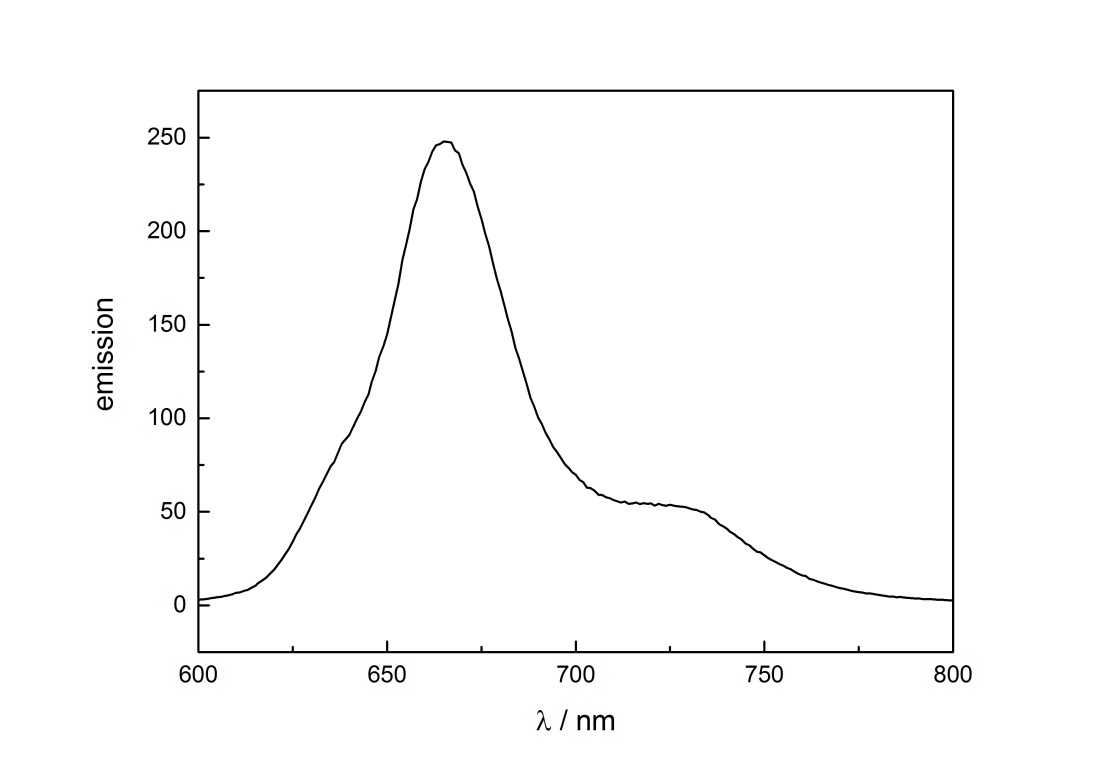


Fig. S6: Emission spectrum of 5, 15-pentafluorophenyl-10-trimethylsilylethynyl-corrole 2.

## Characterization of 5, 15-pentafluorophenyl-10-ethynylcorrole

##
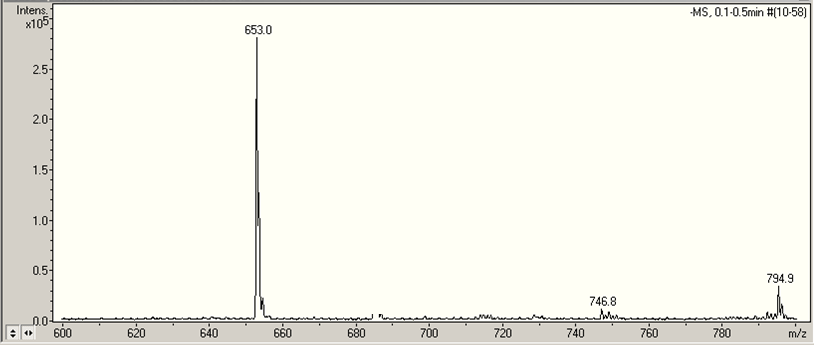


H_3_TpFPC

## Fig. S7: Mass spectrum of 5, 15-pentafluorophenyl-10-ethynylcorrole 3.

Characterization of **5,15-bispentafluorophenyl-10-(pyreneethynyl)corrole**

##
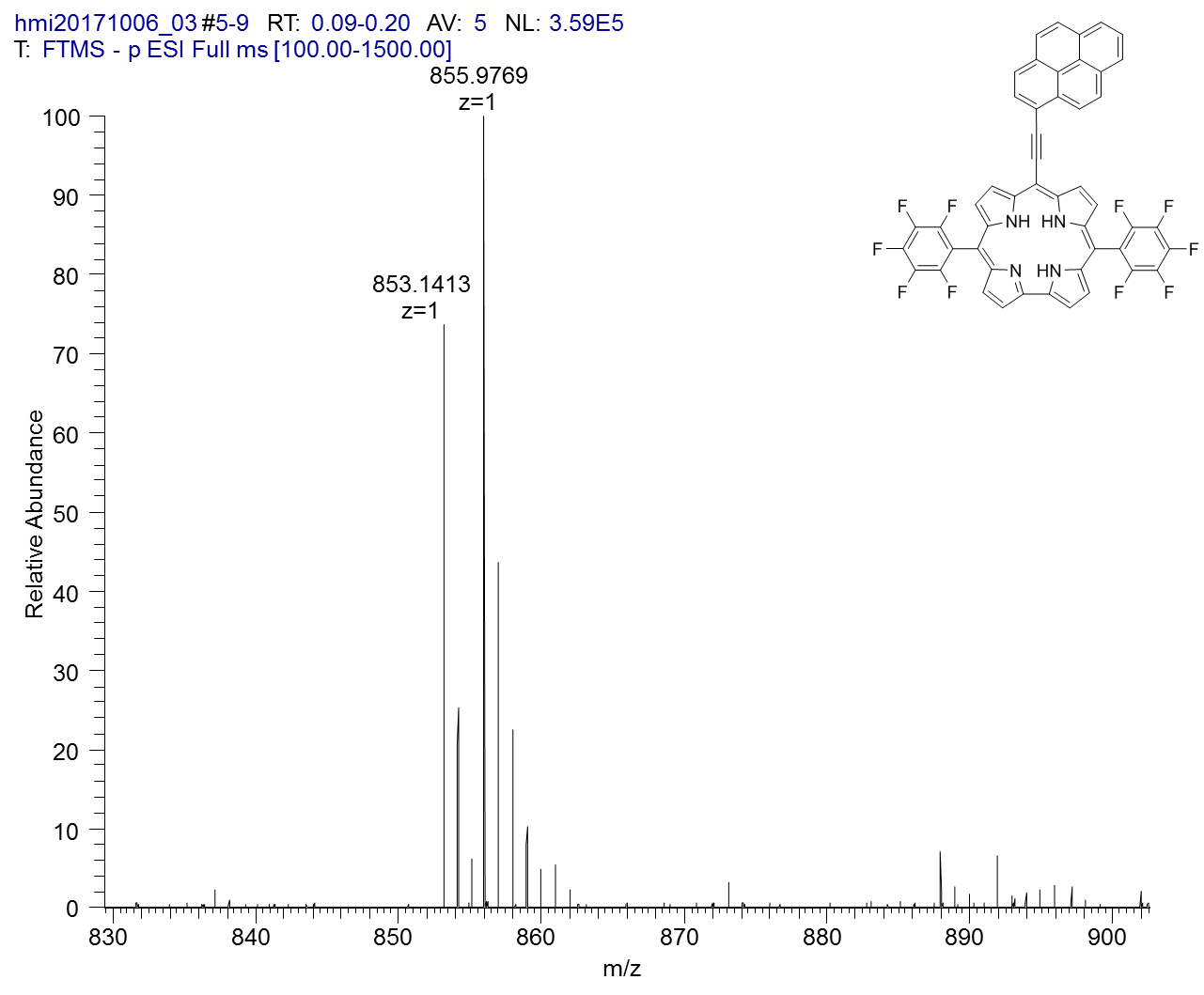


CuTpFPC

Fig S8: HRMS of 5,15-bispentafluorophenyl-10-(pyreneethynyl)corrole**.**

Characterization of copper **5,15-bispentafluorophenyl-10-(pyreneethynyl)corrole**


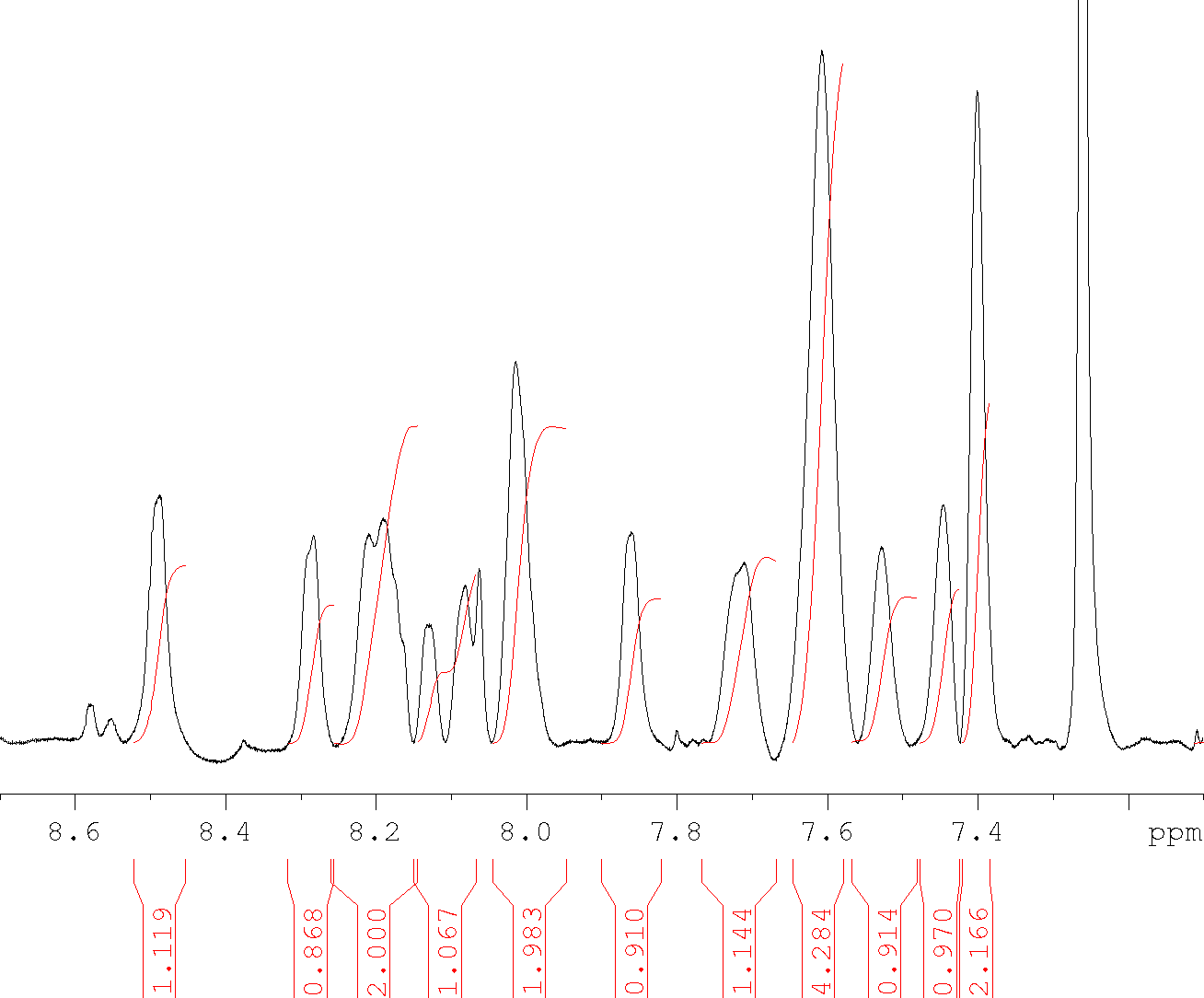


Fig. S9: ^1^H-NMR spectrum of Copper 5,15-bispentafluorophenyl-10-(pyreneethynyl)corrole 4**.**


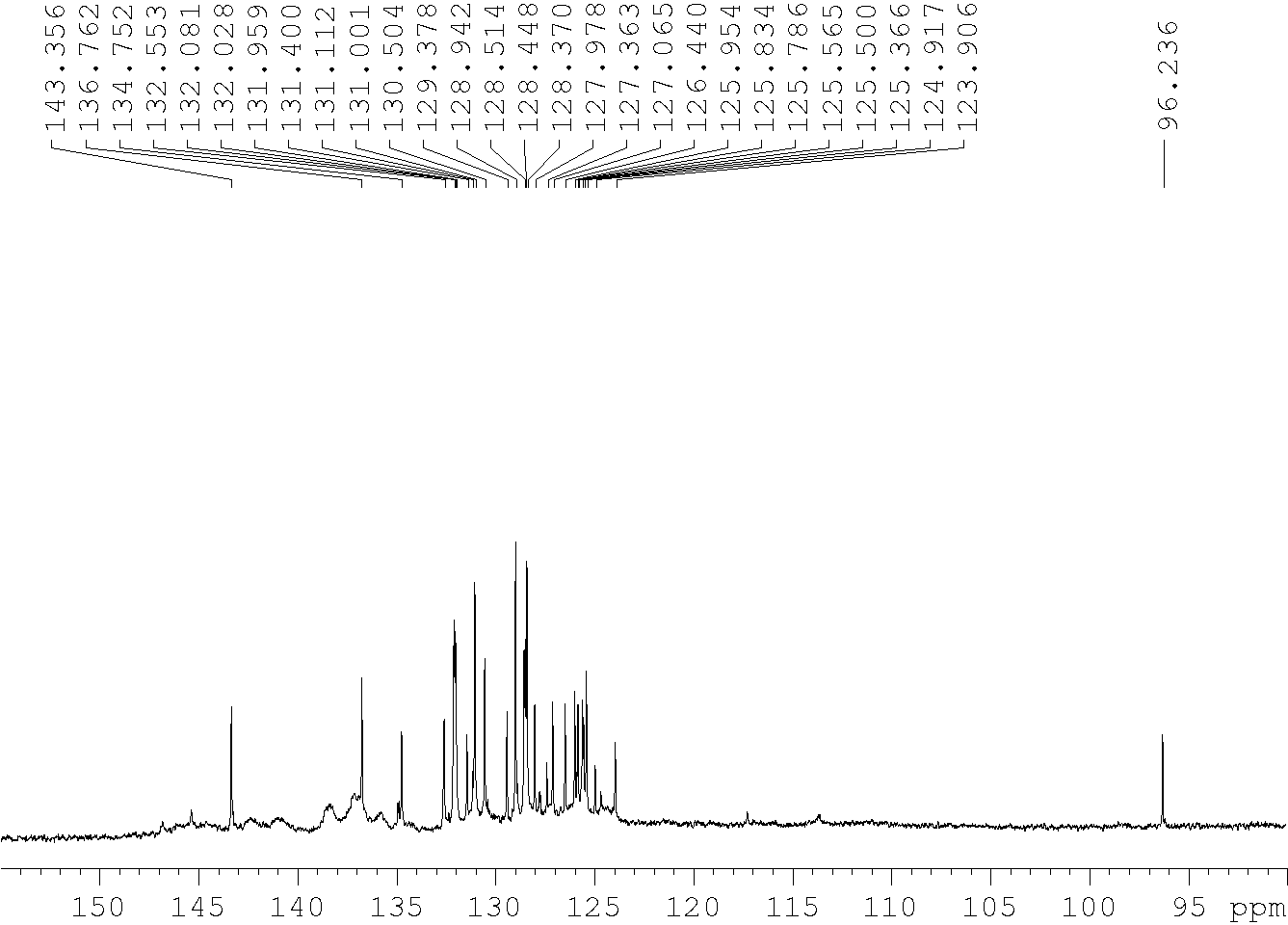


Fig. S10: ^13^C-NMR spectrum of Copper 5,15-bispentafluorophenyl-10-(pyreneethynyl)corrole**.**


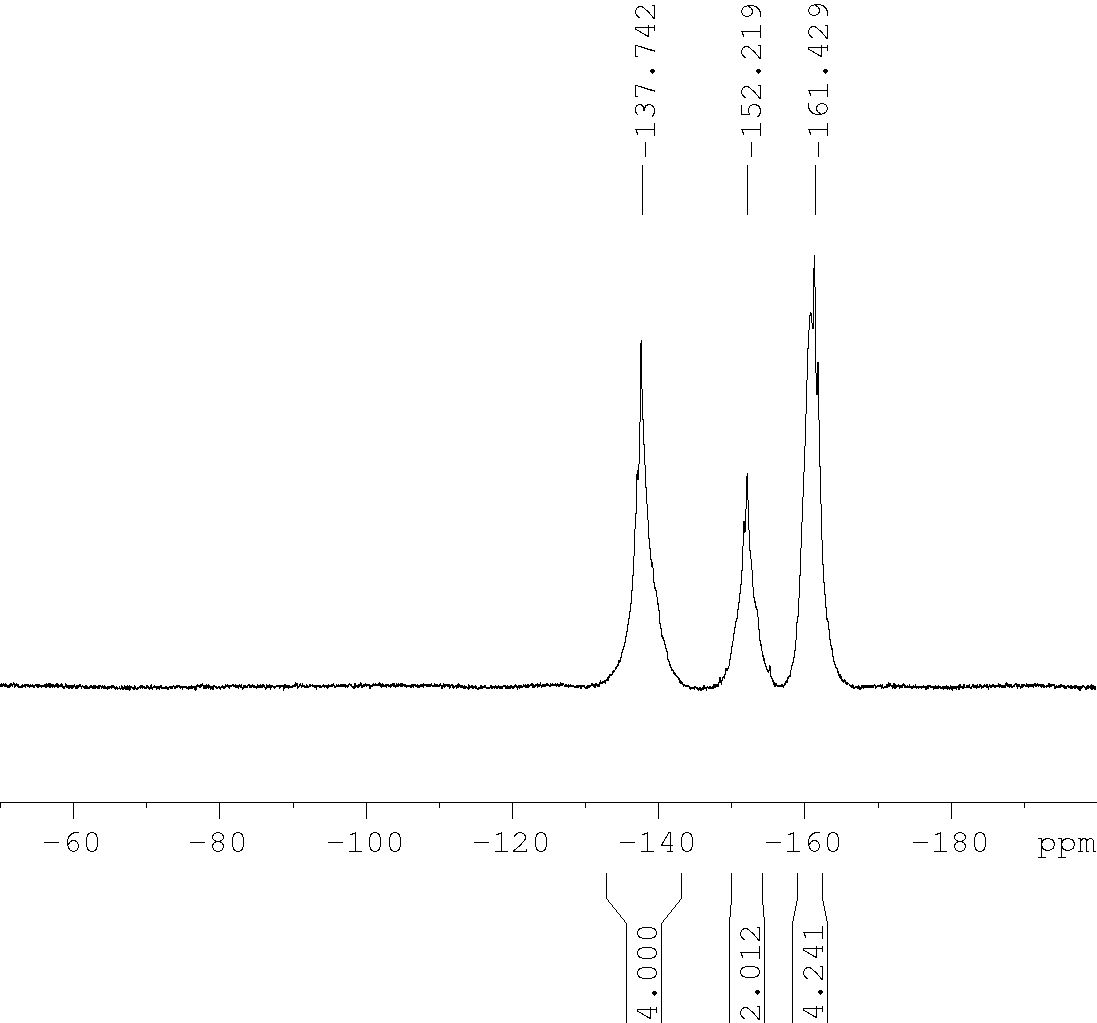


Fig. S11: ^19^F-NMR spectrum of Copper 5,15-bispentafluorophenyl-10-(pyreneethynyl)corrole**.**


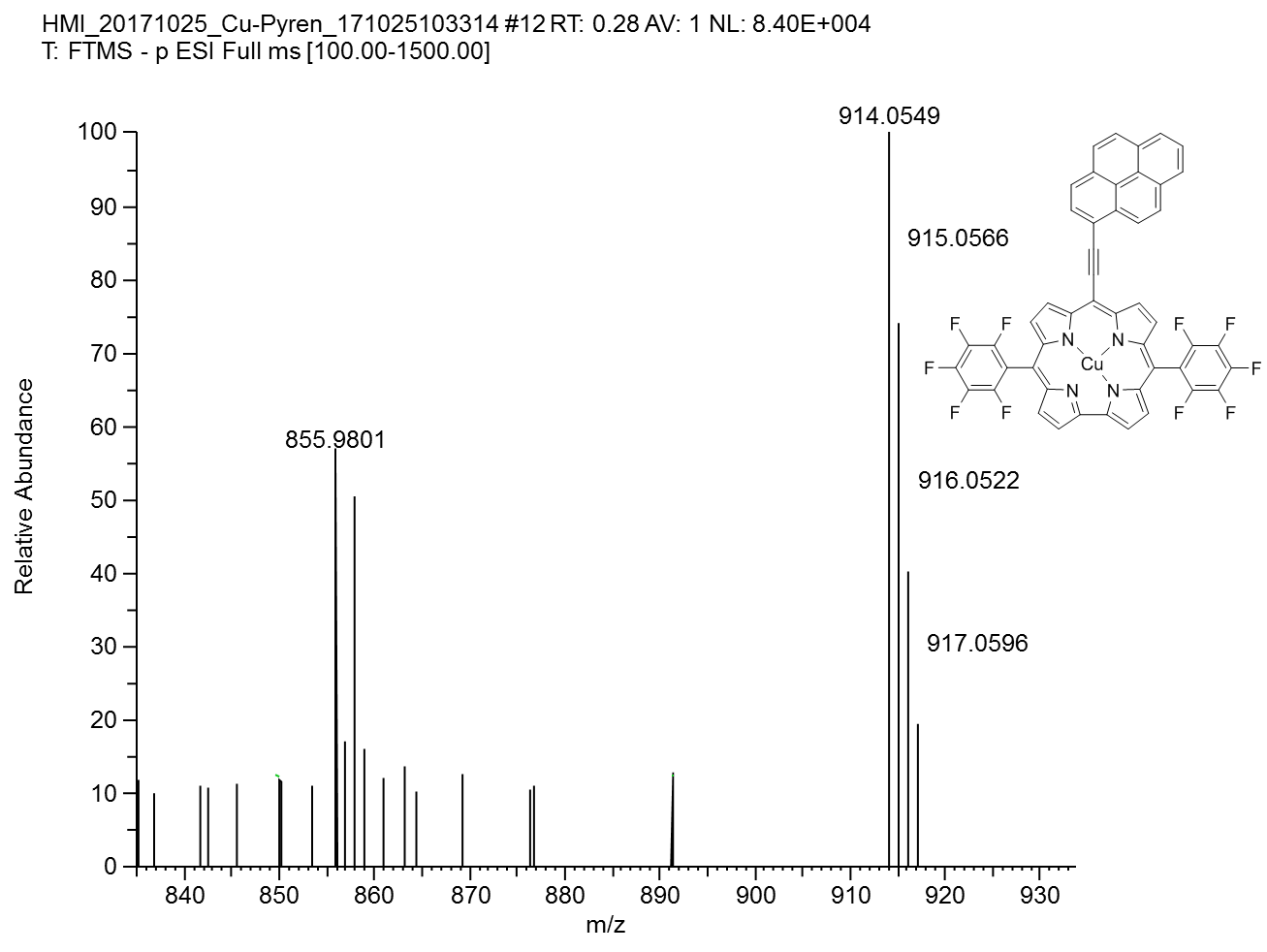


CuTpFPC

Fig S12: HRMS of Copper 5, 15-pentafluorophenyl-10-(pyreneethynyl)corrole (neutral M-peak detected).

[1] R. Orłowski, D. Gryko, D. T. Gryko, *Chem. Rev.* **2017**, *117*, 3102.

[2] M. König, F. Faschinger, L. M. Reith, W. Schöfberger, *J. Porphyrins Phthalocyanines* **2016**, *20*, 96.

[3] J. F. B. Barata, M. G. P. M. S. Neves, M. A. F. Faustino, A. C. Tomé, J. A. S. Cavaleiro, *Chemical reviews* **2017**, *117*, 3192.

[4] a) C. M. Lemon, P. J. Brothers, *J. Porphyrins Phthalocyanines* **2011**, *15*, 809; b) R. Paolesse, *Synlett* **2008**, *2008*, 2215.

[5] a) Z. Gross, N. Galili, *Angew. Chem. Int. Ed.* **1999**, *111*, 2536; b) R. Paolesse, S. Mini, F. Sagone, T. Boschi, L. Jaquinod, D. J. Nurco, K. M. Smith, *Chem. Commun.* **1999**, 1307.

[6] a) I. Aviv-Harel, Z. Gross, *Chem. Eur. J.* **2009**, *15*, 8382; b) R. D. Teo, J. Y. Hwang, J. Termini, Z. Gross, H. B. Gray, *Chem. Rev.* **2017**, *117*, 2711; c) S. M. Borisov, A. Alemayehu, A. Ghosh, *J. Mater. Chem. C* **2016**, *4*, 5822; d) R. Paolesse, S. Nardis, D. Monti, M. Stefanelli, C. Di Natale, *Chem. Rev.* **2017**, *117*, 2517; e) C. M. Lemon, D. C. Powers, P. J. Brothers, D. G. Nocera, *Inorg. Chem.* **2017**, *56*, 10991.

[7] W. Schöfberger, F. Faschinger, S. Chattopadhyay, S. Bhakta, B. Mondal, J. A. A. W. Elemans, S. Müllegger, S. Tebi, R. Koch, F. Klappenberger et al., *Angew. Chem. Int. Ed.* **2016**, *55*, 2350.

[8] M. König, L. M. Reith, U. Monkowius, G. Knör, K. Bretterbauer, W. Schoefberger, *Tetrahedron* **2011**, *67*, 4243.

[9] a) M. Tiffner, S. Gonglach, M. Haas, W. Schöfberger, M. Waser, *Chem. Asian. J.* **2017**, *12*, 1048; b) W. Sinha, M. G. Sommer, N. Deibel, F. Ehret, B. Sarkar, S. Kar, *Chem. Eur. J.* **2014**, *20*, 15920; c) M. Stefanelli, M. Mastroianni, S. Nardis, S. Licoccia, F. R. Fronczek, K. M. Smith, W. Zhu, Z. Ou, K. M. Kadish, R. Paolesse, *Inorg. Chem.* **2007**, *46*, 10791; d) M. Schmidlehner, F. Faschinger, L. M. Reith, M. Ertl, W. Schoefberger, *Appl. Organometal. Chem.* **2013**, *27*, 395.

[10] D. T. Gryko, *Eur. J. Org. Chem.* **2002**, *2002*, 1735.

[11] K. Ueta, K. Naoda, S. Ooi, T. Tanaka, A. Osuka, *Angew. Chem. Int. Ed.* **2017**, *56*, 7223.

[12] H. L. Anderson, *Tetrahedron Letters* **1992**, *33*, 1101.

[13] T. Rohand, E. Dolusic, T. Ngo, W. Maes, W. Dehean, *Arkivoc* **2006**, *2007*, 307.

[14] B. Koszarna, D. T. Gryko, *J. Org. Chem.* **2006**, *71*, 3707.

[15] A. Nowak-Król, R. Plamont, G. Canard, J. A. Edzang, D. T. Gryko, T. S. Balaban, *Chem. Eur. J.* **2015**, *21*, 1488.

[16] G. R. Geier III, B. J. Littler, J. S. Lindsey, *J. Chem. Soc., Perkin Trans. 2* **2001**, 701.

[17] B. J. Littler, Y. Ciringh, J. S. Lindsey, *The Journal of organic chemistry* **1999**, *64*, 2864.

[18] D. T. Gryko, K. Jadach, *J. Org. Chem.* **2001**, *66*, 4267.

[19] Kabeer Ahmed Shaikh, Vishal A. Patil, B.P. Bandgar, *Orbital: Electron. J. Chem.* **2012**, *2012*, 111.

[20] P. Yadav, M. Sankar, X. Ke, L. Cong, K. M. Kadish, *Dalton Trans.* **2017**, *46*, 10014.
